# Correlation between Post-Thaw Spermatozoa Quality of the Endangered Javan Banteng with OPN Gene Expression

**DOI:** 10.1155/2023/9982422

**Published:** 2023-07-05

**Authors:** Vincentia Trisna Yoelinda, Raden Iis Arifiantini, Dedy Duryadi Solihin, Muhammad Agil, Dedi Rahmat Setiadi, Tulus Maulana, Bambang Purwantara, Yohana Tri Hastuti, Jansen Manansang, Dondin Sajuthi

**Affiliations:** ^1^Study Program of Reproductive Biology, School of Veterinary Medicine and Biomedical Sciences, IPB University, Bogor, West Java 16680, Indonesia; ^2^Department of Physiology, Faculty of Veterinary Medicine, Universitas Gadjah Mada, Yogyakarta 55281, Indonesia; ^3^Division of Reproduction and Obstetrics, School of Veterinary Medicine and Biomedical Sciences, IPB University, Bogor, West Java 16680, Indonesia; ^4^Biotech Center, IPB University, Bogor, West Java 16680, Indonesia; ^5^Research Centre of Applied Zoology, National Research and Innovation Agency, Cibinong, Bogor, West Java 16911, Indonesia; ^6^Taman Safari Indonesia Bogor, Cisarua, Bogor, West Java 16750, Indonesia; ^7^Division of Internal Medicine, School of Veterinary Medicine and Biomedical Sciences, IPB University, Bogor, West Java 16680, Indonesia

## Abstract

The role of ex situ conservation facilities or captivity through captive breeding programs is essential in the conservation of the endangered Javan banteng. The development of semen cryopreservation may assist on one side of the conservation plan. However, the male Javan banteng reproductive capability must be considered as it influences the targeted outputs. Studying the potential biomarker for fertility such as osteopontin gene expression is also expected to help predict male fertility. Therefore, this study aimed to analyze the quality of spermatozoa after thawing to help predict the male reproductive capability of Javan banteng. Furthermore, this study investigated the potential role of osteopontin gene expression in male Javan banteng fertility. A positive reinforcement approach was used to accustom the male and female animals as we focused on establishing a collection procedure using neither sedation nor anaesthesia. Semen samples were collected at Taman Safari Indonesia, Bogor, in accordance with the female banteng receptivity. Semen samples were then evaluated and then cryopreserved under field conditions. Our study showed the different predicted reproductive capability of the Javan banteng based on the post-thaw spermatozoa quality, which showed significant differences. The OPN gene showed positive correlations with the progressive motility (*r* = 0.711, *p* = 0.048), viability (*r* = 0.822, *p* = 0.012), and acrosomal integrity (*r* = 0.665, *p* = 0.072) of Javan banteng spermatozoa after thawing. Our study demonstrated the predicted Javan banteng reproductive capability based on various post-thaw spermatozoa variables. This finding is also the first report on the OPN gene potential to be developed as the assessment tool of post-thaw spermatozoa quality of the male Javan banteng. The findings in our study may help give recommendations for future breeding programs, especially in the ex situ conservation sites.

## 1. Introduction

Banteng is experiencing population decline due to various factors and challenges, such as habitat loss, poaching, and conflict with surrounding communities [1, 2]. The Red List of International Union for Conservation of Nature (IUCN) has placed banteng on their list as an endangered species [2]. Banteng is also a priority species in Indonesia according to the Decree of the Director General of Ministry of Environment and Forestry number: 180/IV-KKH/2015. This wild cattle, which is also believed as the ancestor of the Bali cattle [3], is also protected by the Indonesian government based on Ministerial Regulation P.20/MENLHK/SETJEN/KUM.1/6.2018. The strategy and action plan for banteng (*Bos javanicus*) conservation has been established by the government and are written in the Minister of Forestry Regulation No. P.58/MENHUT-II/2011. The global (international) banteng population targeted in that regulation is 300 individuals from 30 breeders without interrupting the wild population. In addition, banteng transfers between conservation facilities are needed to avoid deterioration of the genetic quality and inbreeding. The role of ex situ conservation facilities through captive breeding programs is critical in achieving these targets and strategies.

Spermatozoa cryopreservation can serve as one approach to support this conservation plan. Furthermore, modifications in field conditions are possible [4, 5]. Therefore, this approach can be an option to support the management of captive breeding programs. This approach has several advantages, such as the possibility of exchanging genetic material between conservation sites, captivity or research sites, thereby reducing the level of homozygous in a population [6]. This will help reduce the risk, minimize the cost and time constraint required to transfer banteng to another institution, and avoid the inbreeding depression of the captive banteng population. Packing semen into straws with compact size and clear identification makes it easy to keep and transport banteng genetic material from one conservation facility to another properly.

In addition, cryopreservation of semen allows long-term genetic material storage without significantly compromising the quality. Several studies in closely related species of banteng, which are bovines, have shown a potential shelf life of up to 45 years [7]. In humans, successful IVF has been reported with spermatozoa that have been stored for up to 40 years without any defects in the fetus [8]. This means that it will allow banteng genetic material from a generation to be stored for a long time for long-term captive conservation programs. The work of cryopreservation of wild animal spermatozoa also continues to develop [9, 10]. Several locations, such as San Diego Wildlife Alliance [11], Zoological Park Organization, Thailand, and Taronga Zoo, Australia, have established a bank for their endangered animals' genetic materials [12]. Spermatozoa cryopreservation in wildlife has been previously reported in elephants, pandas, deer, rhinos, leopards, and others [13]. Furthermore, the application of the post-thaw preserved semen for artificial insemination has also been reported to be successfully yielding pregnancy, if not offspring, for example, in pandas [14], elephants [15], and banteng [16].

When exchanging species between conservation facilities, reproductive capability of the animals must also be considered. Essential variables in assessing reproductive capability include age and history of reproductive success [17], the latter is influenced by male fertility. The estimation of the reproductive capability also is very important for the development of suitable assisted reproductive technologies [18]. In addition, the spermatozoa quality varies greatly among wild animals, depending on their species and their nature. Understanding these circumstances is also critical for determining the proper reproductive technology and mitigation to apply. The cryobiological characteristics between species show differences that affect the handling of their cryopreservation [19]. Research on banteng spermatozoa and the development of the assisted reproductive technology is still very limited; meanwhile, the wild banteng population is declining.

Male fertility study with a molecular approach has been growing recently. Various biomarkers have been investigated to see the potency and correlation of genes to the quality of spermatozoa of various species. The osteopontin (OPN) gene is one of the genes studied. However, research on banteng spermatozoa is still limited and uses conventional approaches [20, 21]. Osteopontin is synthesized by the ampullary accessory gland epithelial cells [22], and it is present in seminal plasma and is associated with the freezing ability of bovine semen [23]. Osteopontin is one of the markers showing a consistent correlation with fertility [24]. Osteopontin also acts as a decapacitation factor that prevents premature spermatozoa activation [25]. However, exploration of this gene in banteng has not yet been reported. Thus, our study aimed to assess the quality of post-thaw spermatozoa as a predictor of reproductive capability of Javan banteng. In addition, this study analyzed the potential of the OPN gene expression to be used as a biomarker.

## 2. Materials and Methods

The study was conducted in Taman Safari Indonesia Bogor, Cisarua, West Java, Indonesia. For this study, we avoided the use of sedation or anaesthesia for sampling purposes. We focused on using a positive reinforcement approach for established and sustainable semen collection use in the future. Semen collection methods proposed in this study were artificial vagina and/or transrectal massage. Consequently, there were a limited number of male and female banteng, which was feasible for the following procedure required in this study.

### 2.1. Animal Training and Conditioning to Semen Collection

Animal training and conditioning were conducted to accustom the sample animals to the method of semen collection of choice. The positive reinforcement approach was chosen to minimize stress and discomfort during the habituation process. The male and the female banteng were introduced to different stimuli or instrument step by step. The stimuli used in this study were the sound of a whistle and feed as positive reinforcers or rewards. Another instrument used in giving instructions to each banteng was a target stick, which was gradually replaced with a leash for semen collection purposes with an artificial vagina. The leash was introduced by bringing the leash closer to the banteng's nostril area. Then, the banteng touched the rope of the leash, then subsequently received a reward. The responses of each banteng to the habituation stage were observed and used to determine the collection method to be applied.

Ultimately, two clinically healthy adult male Javan banteng, coded as Banteng 1 and Banteng 2, and one healthy adult female Javan banteng were successfully trained and used for this study. All the animals exhibited no clinical health issue during the study period and were given the same feed such as grass, carrots, and sweet potatoes and were housed individually. All procedures in this study have been approved by the Institutional Animal Care and Use Committee (IACUC), LPPM—IPB University (No: 165–2019 IPB).

### 2.2. Fresh Semen Collection and Evaluation

The method of semen collection was selected based on the results of the habituation carried out beforehand. Banteng 1 was habituated to the artificial vagina method and Banteng 2 to the transrectal massage method. Following the training and conditioning process, successful semen collection was done monthly following the female Javan banteng estrous cycle from May 2021 to August 2021. Each fresh semen sample obtained was then homogenized and immediately evaluated under field conditions to see mass movement, motility, viability, and estimated spermatozoa concentration.

Spermatozoa mass movement and motility were determined subjectively under field conditions. The mass movement was evaluated by examining spermatozoa movement characteristics also known as wave motion from a drop of raw semen sample under the microscope at low magnification (10x). Spermatozoa mass movement scoring was done according to Yendraliza et al. [26]. Spermatozoa motility from each sample was assessed using a brightfield microscope at a magnification of 40x from a minimum of five fields. Examination of spermatozoa viability was carried out by staining with eosin-nigrosin [27]. As much as 10 *μ*l of semen was dropped on a microscope slide, homogenized with eosin-nigrosin solution, and then dried using a heating table. Observations were made at 40x magnification to a minimum of 200 spermatozoa cells. Dead spermatozoa will be stained red or dark pink, while live spermatozoa remain unstained (white) or faint pink. Estimated spermatozoa concentration was evaluated according to Yendraliza et al. [26] by measuring the distance between spermatozoa heads. Spermatozoa concentration was later calculated using Neubauer chamber.

### 2.3. Semen Cryopreservation

Semen cryopreservation was performed for all semen samples obtained, resulting in four batches of samples. The semen extender used was AndroMed® (Minitüb, Tiefenbach, Germany). AndroMed® was mixed with double distilled water according to the manufacturer's protocol. Fresh semen samples collected from each Javan banteng bull were then mixed with the extender. The diluted semen was then packed into 0.25 ml straws and equilibrated for 3 hours at 5°C. Straws were then placed 10 cm above the liquid nitrogen vapor for 10 minutes. Finally, all straws were submerged and stored in a −196°C liquid nitrogen container until further analysis.

### 2.4. Post-Thaw Semen Evaluation

Frozen semen straws were transported using a transport container to the National Research and Inovation Agency (founded as the Indonesian National Research Agency or LIPI), Cibinong, West Java. A total of 16 straws from each banteng bull were examined for progressive motility by computer-assisted sperm analysis (CASA; SpermVision, Germany). Spermatozoa viability was examined with eosin-nigrosine staining. Evaluation of intact acrosome was performed using fluorescens isothiocyanate peanut agglutinin (FITC-PNA) staining and evaluated under the microscope with a green filter.

### 2.5. Osteopontin Gene Primers Optimization

Spermatozoa DNA were extracted using the Quick gDNA Miniprep Kit (Zymo Research, USA) according to the manufacturer's modified protocol. Modifications were carried out by adding mechanical grinding into the protocol to help optimize cell lysis using a micro pestle, then allowed to stand at room temperature for 10 minutes. To amplify the OPN gene (AY878328) in Javan banteng spermatozoa DNA, 1× PCR Buffer (BioLabs, USA), GC Enhancer (BioLabs, USA), dNTP (Qiagen, USA), ddH_2_O, DNA template, forward and reverse primers, and Taq polymerase (BioLabs, USA) were mixed with a final total volume of 25 *μ*L. PCR conditions were as follows: predenaturation at 94°C (3 minutes), then 35 cycles of denaturation at 94°C (30 seconds), annealing at 53°C (30 seconds), 72°C elongation (1 minute), and final extension at. 72°C (5 minutes). The PCR products were visualized on agarose gel and sent to 1st BASE, Malaysia for sequencing.

### 2.6. Quantification of the OPN Gene in Spermatozoa

Prior to the spermatozoa RNA extraction, four batches of each banteng semen straws were thawed at 37°C for 30 seconds. Spermatozoa RNA extraction was performed using Direct-zol RNA Miniprep Kits (Zymo Research, USA) according to the manufacturer's protocol. Ribonucleic acid (RNA) from each sample was then synthesized into complementary DNA (cDNA) using the SensiFAST™ cDNA synthesis kit (Meridian Bioscience, USA). Quantitative real-time polymerase chain reaction (qRT-PCR) was carried out with both the OPN gene and the peptidylprolyl isomerase A (PPIA) gene as the housekeeping gene [28] using SensiFAST™ SYBR® mix (Meridian Bioscience, America). Amplification using CFX Opus 96 Real-Time PCR System (Bio-Rad, USA) with annealing at 55°C for the PPIA gene and 53°C for the OPN gene. The value of delta Ct (Δ*Ct*) was calculated as the difference between the target gene and the reference gene. Relative expression was calculated based on Ct according to the formula of Livak and Schmitgen [29]. All procedures were performed at the Laboratory of Biotechnology, Primate Research Center, Institution of Research and Community Services (LPPM)—IPB University, Lodaya, Bogor, West Java. The details of primer designs used in this study are listed in Table 1.

### 2.7. Data Analysis

Fresh semen and post-thaw spermatozoa quality were presented as the mean and standard deviation. Normality and equality of variances for each variable were tested first. The normally distributed data with the same variances were then tested with the Student's T-test, while the Mann–Whitney nonparametric test evaluates data that are not normally distributed. The correlation between the relative expression value of the OPN gene and the post-thaw spermatozoa quality variable was tested by Pearson correlation analysis.

## 3. Results and Discussion

### 3.1. Results

#### 3.1.1. Training and Conditioning to Semen Collection

Banteng 1 and female banteng were successfully trained and conditioned to the leash and can be tethered in the service crate in the semen collection area. The temperament of Banteng 1 in a special area for semen collection was relatively calm, making it easier for zookeepers to guide the animals for the artificial vagina method. However, Banteng 1 was relatively restless when in the modified squeeze chute. Banteng 2 showed a calmer temperament when herded into the modified squeeze chute. The difference in temperament and the banteng bull's response to stimuli during habituation became the basis for determining the semen collection method for each individual. Banteng 1 was successfully habituated to semen collection using an artificial vagina. Meanwhile, Banteng 2 was habituated to semen collection using the transrectal massage method. The female banteng required four months of training and conditioning to be used as a teaser for the semen collection using the artificial vagina. The temperament of the female banteng used in our study was calmer than that of other female individuals based on observations and secondary data. The habituation took approximately four to six months until the animals were accustomed to the procedures. It differed between the individual according to their response to the procedures.

#### 3.1.2. Fresh Semen Quality

Five attempts were made to collect semen samples from Banteng 1 and Banteng 2, resulting in four semen batches. The fresh semen quality of each banteng is shown in Table 2. There were significant differences in the mass movement and motility (*p* < 0.05) among the two banteng bulls. Nevertheless, the spermatozoa viability showed no significant difference.

#### 3.1.3. Post-Thaw Spermatozoa Quality

The progressive motility and viability of post-thaw spermatozoa evaluated from 16 straws from each banteng showed significant difference between Banteng 1 and Banteng 2, whereas the quality of post-thaw spermatozoa of Banteng 1 showed significantly better quality compared to Banteng 2. The quality of semen after thawing is shown in Table 3.

#### 3.1.4. Osteopontin Gene Analysis

**Figure 1:**
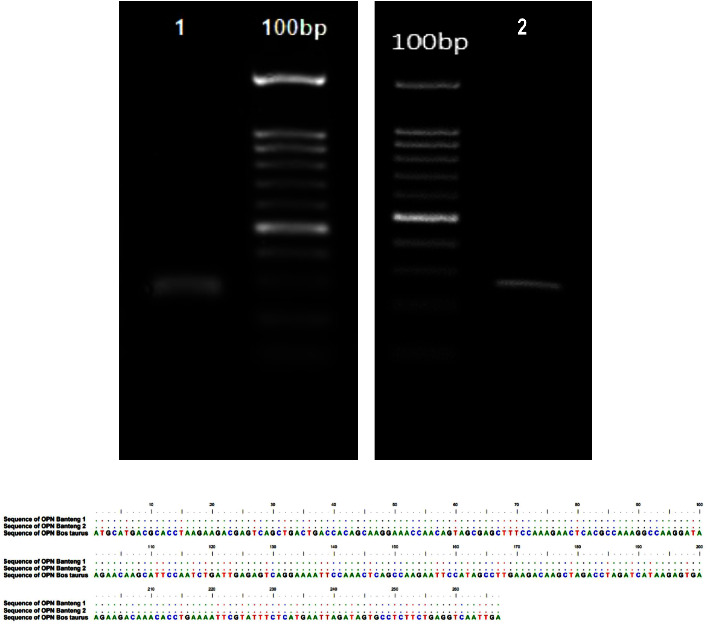
(a) Visualization of approximately 267 bp PCR product on agarose gel. (b) The sequences of Banteng 1 and Banteng 2 OPN gene aligned to *Bos taurus* (AY878328) sequence.

The OPN gene was successfully amplified in Banteng 1 and Banteng 2 spermatozoa samples (Figure 1). Sequence alignment results showed that the primer bound well to the template and produced a sequence of 267 bp.

**Figure 2:**
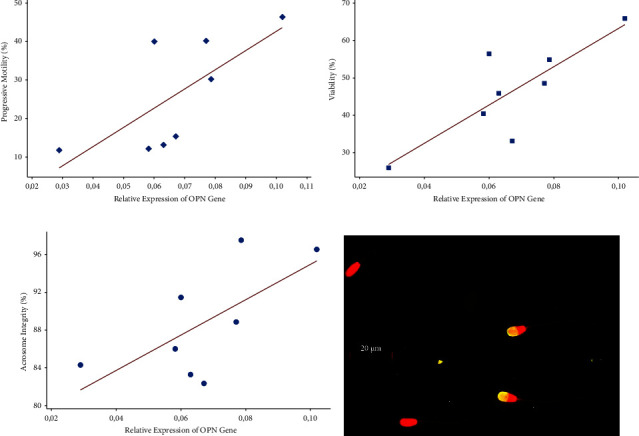
Correlation graph of the relative gene expression with the evaluated post-thaw spermatozoa variables. (a) Positive correlation between OPN gene with spermatozoa progressive motility. (b) Positive correlation between OPN gene with spermatozoa viability. (c) Positive correlation between OPN gene with spermatozoa acrosomal integrity. (d) Visualization of spermatozoa with and without intact acrosome, note that intact acrosomes were stained in bright yellow fluorescent.

The OPN gene in this study showed positive correlations to progressive motility (*r* = 0.711, *p* = 0.048), viability (*r* = 0.822, *p* = 0.012), and acrosomal integrity (*r* = 0.665, *p* = 0.072) (Figure 2). Spermatozoa with intact acrosome were displayed with bright yellow fluorescent color on the anterior part.

## 4. Discussion

Prediction on animal reproductive capabilities plays an important role in the breeding management program. It is helpful in ensuring that the selected management program is feasible. To increase the population size and for population continuity, it is necessary to ensure the availability of individuals with qualified reproductive capabilities. A model presented by Manlik et al. [30] showed that population viability is strongly influenced by reproductive ability. The higher the variation in the reproduction rate, the greater the impact on population viability.

Implementing spermatozoa cryopreservation as one of the conservation strategies also goes through the semen collection stage, which is challenging in several wildlife animals. Several methods of semen collection in wild animals apply anaesthetic procedures for restraining and handling purposes. Another approach that has been reported is the habituation process based on positive reinforcement. The process of habituation or training and conditioning with positive reinforcement is represented by the use of associations between stimuli followed by rewards, which may be in the form of feed as a positive reinforcer. The habituation process for semen sample collection has been carried out on various wildlife, such as elephants, yaks, and rhinos [31–34]. Factors that must be considered in the habituation or training process are the animal size, its temperament and the equipment for semen collection [35]. The Javan banteng individuals used in our study had a calm temperament and could therefore be trained in a relatively short time. Previous studies have shown that there are a variety of differences in the timing and approaches to semen collection. In addition, the availability of semen collection equipment also varies among the institutions. Taman Safari Indonesia Bogor (TSI Bogor), the site where our study was conducted, fortunately, has owned and established several crucial devices and set up related to semen collection. The equipment available at TSI Bogor already supports semen collection using the artificial vaginal method and transrectal massage method.

The quality of the fresh semen used affects the outcome of the post-thaw quality. However, in our study, the entire sample was used for cryopreservation as a preliminary study and the subsequent research stage. Johnston et al. [16] reported the mass movement in fresh banteng semen with a score of three. Therefore, our study's results were still below those of previous studies. Although not all the variables evaluated in the study showed significant differences between the two banteng bulls, the value of progressive motility and viability of the Javan banteng spermatozoa in this study were still below the results of previous studies by Johnston et al. [16] and Yoelinda et al. [36].

During preservation, spermatozoa undergo physical and biochemical changes [37]. Those changes will affect spermatozoa quality, such as motility and viability [38]. Bovine spermatozoa are very sensitive to cryopreservation [39], which can be indicated by decreased viability, motility, and post-thaw spermatozoa movement scores. Javan banteng spermatozoa were reported to show similar characteristics to bovine spermatozoa [40, 41]. A decrease in sperm motility and viability also has been reported up to 50% compared to the fresh semen quality as reported elsewhere [38, 42, 43]. As the quality of fresh semen differed between Banteng 1 and Banteng 2, the quality of post-thaw spermatozoa also showed different results. Our result is in agreement with the previous study by Nagata et al. [44] that showed the variation of spermatozoa cryosurvival, resulting in variation in the post-thaw spermatozoa quality. Therefore, it was recommended to take fresh semen spermatozoa quality into account prior to cryopreservation. Moreover, according to the record, there was no difference in the feeding and care management of Banteng 1 and Banteng 2. It could be assumed that the spermatozoa quality difference in our study was more likely due to individual factors.

The results of this study indicated a high variation in the predicted reproductive capability of the Javan banteng although both samples have reached sexual maturity. The post-thaw progressive motility of the Banteng 1 spermatozoa was close to the minimum frozen semen standard set by the Indonesian National Standards Agency, which is 40%. However, this value is still below the previously reported data, which is 45% [16]. Spermatozoa motility after thawing is an essential factor for the success of artificial inseminations in various animals. The post-thaw viability of Banteng 1 and Banteng 2 spermatozoa were lower than in yaks (70.40%) [45] and Bali cattle (64.69%–71.23%) [46]. Research on other wildlife animals also showed varied results. For example, in the Sunda clouded leopards, it was only 13.60% and 11.60% [47], 47.05% for Javan leopard [48], whereas in the rhino, it reached 91.40% [49].

Cryopreservation can also affect the acrosomal membrane and induce a preacrosomal reaction, thus affecting the viability and quality of spermatozoa. The acrosome is a hood-like structure covering the spermatozoa's anterior part. The acrosome area of spermatozoa contains various proteins that play an essential role in the acrosome reaction and penetration with the zona pellucida [50]. Acrosomal integrity is important to prevent premature acrosomal reactions and low acrosomal integrity has been associated with infertility [51]. According to Bailey et al. [52], freeze-thawing causes capacitation-like reactions in mammalian spermatozoa. Interestingly, the value of spermatozoa acrosomal integrity in our study was relatively high compared to several species that had been previously reported. The percentage of post-thaw acrosomal integrity in rhinos was reported to be 80.00% [49] and 68.00% [53], whereas in bison it was 71.13%–76.56% [54]. Here, we also noticed that spermatozoa acrosomal integrity in both samples used in our study showed a considerably high percentage regardless of the low post-thaw spermatozoa motility and viability. A similar pattern has been reported in [42], showing a smaller percentage of decrease in sperm acrosomal integrity occurred due to cryopreservation. Acrosomal damage in cryopreserved spermatozoa was also reported to be minimal, around 10%, according to Khalil et al. [38].

The latest research on various species has also been progressing into the “omics” exploration to look for biomarkers to support reproduction. One of the “omics” approaches studied is transcriptomic, which describes the mRNA transcripts content in the spermatozoa [55, 56]. It has been suggested that various gene transcripts may have a potential impact on fertility, one of which is osteopontin (OPN). The presence of OPN has been detected in the reproductive organs of various animals, such as in cattle [22], pigs [57], and even in buffalo seminal plasma [58]. Research by Bustamante-Filho et al. [59] showed that OPN was more highly expressed in the accessory gland fluid of high-fertility bulls than in low-fertility bulls. Other studies showed that OPN improves *in vitro* fertilization outcomes. Osteopontin supplementation during IVF increased the penetration rate, reduced polyspermy incidence, and increased the efficiency of monospermic fertilization [60]. Osteopontin also plays a positive role in acrosomal reaction and capacitation [61] and may have a role in the spermatozoa-oocyte interaction and fertilization [62].

OPN gene expression analysis showed that the OPN gene was expressed in Banteng 1 and Banteng 2 spermatozoa samples. Cancel et al. [22] found that the OPN in bovine seminal plasma originated from the vesicular gland and ampulla. The detection of the target gene transcripts in spermatozoa samples may indicate that the vesicular glands and ampulla of the Javan banteng produce OPN in the seminal plasma that is eventually bound to the spermatozoa membrane. Research on mithun has shown the essential role of OPN in membrane stability. It results in a better resistance of spermatozoa to cryopreservation [21].

Our study also showed that OPN was positively and significantly correlated with post-thaw motility. A similar result has been reported in pigs [63]. Furthermore, the research elsewhere [64, 65] also demonstrated the association of the OPN gene on spermatozoa motility. The high correlation value between OPN and post-thaw spermatozoa viability shown in our study may be related to the antiapoptotic and effects of OPN on cell survival through various mechanisms as reported elsewhere [66, 67]. A positive correlation between OPN and the spermatozoa acrosomal integrity was also found in our study. Osteopontin transcripts expressed in the adluminal compartment of the seminiferous tubules may play a role in the development of elongated spermatids. In their study, Souza et al. [62] found that OPN transcripts are significantly more highly expressed in the acrosomal region of ejaculated spermatozoa. They may also aid vesicle aggregation during acrosome formation [68].

Our study is the first report on the predicted reproductive capability of Javan banteng inferred based on the post-thaw spermatozoa quality and their correlations with the OPN gene transcripts. Based on the overall results aforementioned in our study, we may suggest that the OPN gene has a potential role in the quality of post-thaw spermatozoa, as in the study of Rego et al. [23]. However, further research still needs to be conducted. It is important to note that Javan banteng in our study showed significantly different predicted reproductive capabilities. In this case, differences in semen collection methods might play a role. However, further studies regarding the specific factors and causes are still needed. This also implies that in order to establish a captive breeding program especially using a semen cryopreservation approach in wild animals, the condition and temperament of the animals for semen collection, the facilities available, as well as the manpower must be taken into consideration. Despite the limitations, our study may help in arranging and establishing future breeding programs, especially in the ex situ conservation sites. It can also be useful for the Javan banteng future conservation plan suggestion and improvement.

## 5. Conclusions

In conclusion, we present the first report on the Javan banteng various post-thaw spermatozoa variables and their correlation with the OPN. Our study suggests that the OPN gene expression has the potential to be developed as the assessment approach to predict the reproductive capability of male Javan banteng. The interaction and mechanism of action of this gene in affecting post-thaw spermatozoa still need to be elucidated. However, the limited number of adult Javan banteng feasible for habituation and semen collection is inevitable in this study. It is also important to note that the individual response to the habituation differed in each Javan banteng and resulted in different outcomes. In addition, best practices for the semen collection method must be further studied and established to overcome the challenges implied in our study.

## Figures and Tables

**Table 1:** Genes used for validation and quantification in this study.

Gene	Primer sequence	Accession ID	Annealing temperature
OPN	F: 5′-ATGCATGACGCACCTAAGAAG-3′ R: 5′-TCAATTGACCTCAGAAGAGGC-3′	AY878328	53°C

PPIA	F: 5′-ATGCTGGCCCAACACAA-3′ R: 5′-CCCTCTTTCACCTTGCCAAA-3′	XM_001252921.1	55°C

**Table 2:** Fresh semen quality of Javan banteng.

Variables	Banteng 1	Banteng 2
*Mass movement*	++	—
Spermatozoa motility (%)	66.25 ± 2.5^a^	35.00 ± 16.83^b^
Spermatozoa viability (%)	69.06 ± 5.75^a^	63.82 ± 4.72^a^
Spermatozoa concentration (10^6^ cells/ml)	Semi densum (716.30 ± 19.40)	Semi densum (646.50 ± 66.30)

**Table 3:** Post-thaw spermatozoa quality of Javan banteng.

Variables	Banteng 1	Banteng 2
Spermatozoa progressive motility (%)	39.11 ± 2.08^a^	13.05 ± 2.08^b^
Spermatozoa viability (%)	57.16 ± 7.91^a^	36.27 ± 8.71^b^
Spermatozoa acrosomal integrity (%)	93.55 ± 3.26^a^	83.97 ± 3.92^b^

## Data Availability

The data used to support the findings of this study are available within the article. Further inquiries, such as the raw datasets requests, can be directed to the corresponding author.
